# Differential effects of altered patterns of movement and strain on joint cell behaviour and skeletal morphogenesis

**DOI:** 10.1016/j.joca.2016.06.015

**Published:** 2016-11

**Authors:** L.H. Brunt, R.E.H. Skinner, K.A. Roddy, N.M. Araujo, E.J. Rayfield, C.L. Hammond

**Affiliations:** † The School of Physiology, Pharmacology and Neuroscience, Biomedical Sciences, University of Bristol, BS8 1TD, UK; ‡ School of Earth Sciences, Life Sciences Building, University of Bristol, BS8 1TQ, UK

**Keywords:** Zebrafish, Biomechanics, Joint morphogenesis, Cells, Hypermobility, Paralysis

## Abstract

**Objective:**

There is increasing evidence that joint shape is a potent predictor of osteoarthritis (OA) risk; yet the cellular events underpinning joint morphogenesis remain unclear. We sought to develop a genetically tractable animal model to study the events controlling joint morphogenesis.

**Design:**

Zebrafish larvae were subjected to periods of flaccid paralysis, rigid paralysis or hyperactivity. Immunohistochemistry and transgenic reporters were used to monitor changes to muscle and cartilage. Finite Element Models were generated to investigate the mechanical conditions of rigid paralysis. Principal component analysis was used to test variations in skeletal morphology and metrics for shape, orientation and size were applied to describe cell behaviour.

**Results:**

We show that flaccid and rigid paralysis and hypermobility affect cartilage element and joint shape. We describe differences between flaccid and rigid paralysis in regions showing high principal strain upon muscle contraction. We identify that altered shape and high strain occur in regions of cell differentiation and we show statistically significant changes to cell maturity occur in these regions in paralysed and hypermobile zebrafish.

**Conclusion:**

While flaccid and rigid paralysis and hypermobility affect skeletal morphogenesis they do so in subtly different ways. We show that some cartilage regions are unaffected in conditions such as rigid paralysis where static force is applied, whereas joint morphogenesis is perturbed by both flaccid and rigid paralysis; suggesting that joints require dynamic movement for accurate morphogenesis. A better understanding of how biomechanics impacts skeletal cell behaviour will improve our understanding of how foetal mechanics shape the developing joint.

## Introduction

We now understand that the mechanical environment experienced during early development is important for normal skeletal development. There are multiple conditions for which abnormal or reduced movement are causal; including developmental dysplasia of the hip (DDH), which affects 1.3 per 1000 births^1^, ^2^, arthrogryposis which affects around 1:4000 births^3^ and fetal akinesia deformation sequence (FADS) which affects 1:15,000 births^4^, ^5^. Additionally, there is evidence that early changes to joint shape lead to osteoarthritis (OA) later in life^6^. This can arise if conditions such as DDH are uncorrected^7^, but also subtle changes to hip shape have been identified as conferring increased risk of OA^8^, ^9^. Despite the clinical significance of joint shape, relatively little is known about the underlying cellular events underpinning joint morphogenesis^10^, ^11^.

Many studies have investigated the effect of temporal paralysis on joint formation. The majority have been undertaken in developing chick and mouse limbs and have shown that paralysis caused flattening of articular surfaces and a failure of joint cavitation, resulting in fusion of opposing elements^12^, ^13^, ^14^, ^15^. By contrast, few studies have focused on the role of biomechanics in craniofacial development, though craniofacial morphogenesis is also affected by paralysis, with different joints differentially affected in chicks^13^, ^16^, ^17^. It is less clear what the effect of more sustained hyperactivity of the system will be and whether this would be beneficial to the skeletal system; for example, in chick, the effects of hypermobility have been described to increase joint cavity size^18^.

Previously it was believed that joint morphology developed after cavitation, however, recent studies of chick knee and hip joints have revealed that morphogenesis precedes cavitation, with most anatomical features present prior to element separation^19^, ^20^, lineage tracing in mouse also reveals morphogenesis prior to separation^21^.

We still know relatively little about the cellular events that underpin morphogenesis; though recent work has started to address this question. In chick knee development, patterns of mechanical strain co-localise with regions of increased cellular proliferation, giving clues that *in vivo* cellular behaviour is altered mechanically^22^. In zebrafish, movement is required for normal chondrocyte intercalation^23^ and correct cell orientation at the joint^24^. Recently, there has been increased focus on identifying putative mechanosensitive genes that could couple mechanical forces to downstream morphological responses^14^, ^25^, ^26^. Zebrafish, with their many transgenic lines marking various cell types of the musculoskeletal system^27^ raise the prospect of using imaging to help unravel the cellular dynamics that underpin skeletal morphogenesis. We wanted to compare the effects of rigid paralysis and hyperactivity with flaccid paralysis and observe their impact on jaw joint morphology.

## Materials and methods

### Zebrafish husbandry

Zebrafish were housed as previously described^28^. Animal experiments were ethically approved by the local ethics committee and by the Home Office.

### Pharmacological treatment

Fish were treated from 3 days post fertilisation (dpf) to 5 dpf, with drugs replaced twice daily diluted in Danieau buffer in petri dishes^28^. Flaccid paralysis anaesthetic MS222 (Tricaine methanesulfonate), (Sigma) was used at 0.1 mg/ml. Decamethonium bromide (DMB) induces rigid paralysis and has been used to induce paralysis in chicks in ovo leading to alterations to joint patterning^12^, ^13^, ^14^. DMB was used at 8 mg/ml diluted into Danieau buffer. 4-amino-pyridine (4-AP), a potassium channel antagonist can induce hyperactivity in chick foetuses^29^. 4AP was tested at concentrations from 0.05 mM to 1.2 mM and 0.5 mM was selected for further analysis.

### Tracking of fish swim motility

Swim motility was measured by tracking individual control or 4AP-treated fish from movies. Tracking was performed using a manual tracking ImageJ plugin^30^, which when calibrated for pixel size and time interval between frames allows quantification of distance travelled and velocity (Sup. Vid. 1). Measurements were made on 10 fish per 4-AP dose per time period of drug application.

Supplementary data related to this article can be found online at http://dx.doi.org/10.1016/j.joca.2016.06.015

The following are the Supplementary data related to this article:

Supplementary Video 1 Zebrafish swim motility tracking. Control and 4AP-treated zebrafish swim motility was recorded in petri dishes. ImageJ software was used to manually track the distance of zebrafish movement in 6 min measured from the end of each larval tail. Velocity of zebrafish movement could then be determined from the distance travelled over time.

### Recording frequency of jaw movement

Zebrafish were anaesthetised with MS222 and mounted laterally onto coverslips in 1% agarose. The agarose surrounding the head was removed and Danieau buffer flushed over the coverslip until jaw movements resumed. The number of mouth movements per minute was recorded from four fish per timepoint with 3 measurements taken per fish and mean values used (Sup. Vid. 2). 2-tailed students *t*-tests were used to compare control with 4AP-treated larvae and siblings with *vhl* mutants.

Supplementary data related to this article can be found online at http://dx.doi.org/10.1016/j.joca.2016.06.015

The following are the Supplementary data related to this article:

Supplementary Video 2 Recording of frequency of jaw movement. Brightfield video of a 4dpf larva embedded in 1% agarose and bathed in Danieau buffer with agarose removed from around the head to allow for jaw movements.

### Measurement of jaw displacement

High-speed movies were made of jaw movements in wild type and *vhl* mutants; frames corresponding to maximum jaw displacements were selected, imported into ImageJ^31^ and measurements taken on the distance between the tip of the Meckel's cartilage (MC) in the lower jaw and upper jaw in μm (Sup. Fig. 1). Two-tailed Student *t* test was used to compare average displacements from *vhl* mutants to controls.

### Zebrafish lines

All transgenic and mutant lines have been previously described: Col2a1:mcherry^32^, ^33^; myod^fh261^^34^; VHL *vhl*^*hu2117*^^35^; *Tg(-4725sox10:GFP)*^*ba*4^^36^; symhc:GFP^37^. *Vhl* mutants, in which Hif2a is stabilised, display a hypoxic response despite being well oxygenated^35^, ^38^.

### Wholemount immunohistochemistry

Immunohistochemistry was performed as previously described^24^. Larvae were fixed in 4% PFA and stored in 100% MeOH, rehydrated into PBS with 0.1% Tween (PBSTw), permeabilised using 15 μg/ml proteinase K, washed and blocked in PBS+ 5% horse serum for at least 2 h. The larvae were incubated with anti-myosin A4.1025 mouse IgG [1:200 dilution; Developmental Studies Hybridoma Bank (DSHB)] or rabbit anti-col2 IgG (1:500 dilution, Abcam) in blocking solution overnight at 4°C and washed a minimum of four times in 1×PBSTw. Larvae were incubated with secondary antibodies (Dylite 488 goat anti-mouse IgG and Dylite 550 goat anti-rabbit IgG, Molecular Probes, 1:500 dilution) then washed extensively in 1×PBSTw prior to visualisation. Controls were exposed to only secondary antibodies.

### Analysis of shape variation

Changes to MC shape caused by flaccid paralysis (MS222, *Myod*), rigid paralysis and hyperactivity were quantified using two-dimensional (2D) geometric morphometrics. 2D MC outlines [Sup. Fig. 2(A)] of 5dpf controls (*n* = 15), *vhl* mutants (*n* = 4), *Myod* mutants (*n* = 8) were prepared (Adobe Illustrator) and compared with outlines from larvae treated from 3-5dpf with MS222 (*n* = 18) and DMB (*n* = 4). The outlines were converted into 200 XY coordinates with a common origin located at the anterior tip using TpsDig 2.25^39^ [Sup. Fig. 2(B)]. Coordinates were converted to sine and cosine components using Hangle Fourier transformations^40^ and superimposed using Procrustes superimposition. To assess shape variation qualitatively, the data were subjected to a between groups principal components analysis (PCA) and one-way non-parametric MANOVA. The analyses were performed using Paleontological statistics software (Past 2.17)^41^.

### Finite element models

The meshes for the FE models have been previously described^24^. Loads relating to the Protractor hyoideus (PH), Adductor Mandibulae (AM) and Intermandibularis were applied simultaneously to model rigid paralysis. The FE results are displayed as colour-coded strain contour plots to compare spatial distribution of tension and compression and their magnitude.

### Cell orientation, area and shape

Z-projections of 2–3 slices were created from images of MC chondrocytes labelled with Type 2 collagen. Individual cells were identified and selected by adjusting the threshold of the image, using Image J^31^. Measurements of cell area, length of the major and minor cell axis and angle of the longest axis of each cell were taken for cells at the MC. Major/minor axis was calculated to determine the cell circularity, with 1.00 indicating a perfect circle. The angle of the longest axis of the cell was adjusted in relation to the jaw midline. Graphs were produced and statistics performed using SPSS software (Version 23). A minimum of 9 cells located between the insertion points of the intermandibularis muscle were taken per fish with 3 fish used for each experimental condition.

### Gap analysis

The interval between the MC and PQ cartilage elements of the jaw joint on their medial and lateral sides (typically the smallest and largest gaps between cartilage across the joint, respectively) were measured from confocal images using Leica LAS AF Lite software. Negative values were recorded for overlapped cartilage elements.

Kruskal–Wallis tests (used to make multi-comparisons between non-normal data) were performed to compare joint gap size.

### Microscopy

Live zebrafish were mounted ventrally on coverslips in 1% low melting point agarose containing MS222. A confocal stack was produced by taking images at 1.6 μm intervals through the ventral jaw of 5dpf zebrafish carrying the *Tg(Col2a1aBAC:mcherry)* transgenic reporter or larvae immunostained for type II collagen using an SP8 or SP5 Leica confocal microscope using LAS capture software.

## Results

### Induction of paralysis

**Fig. 1:**
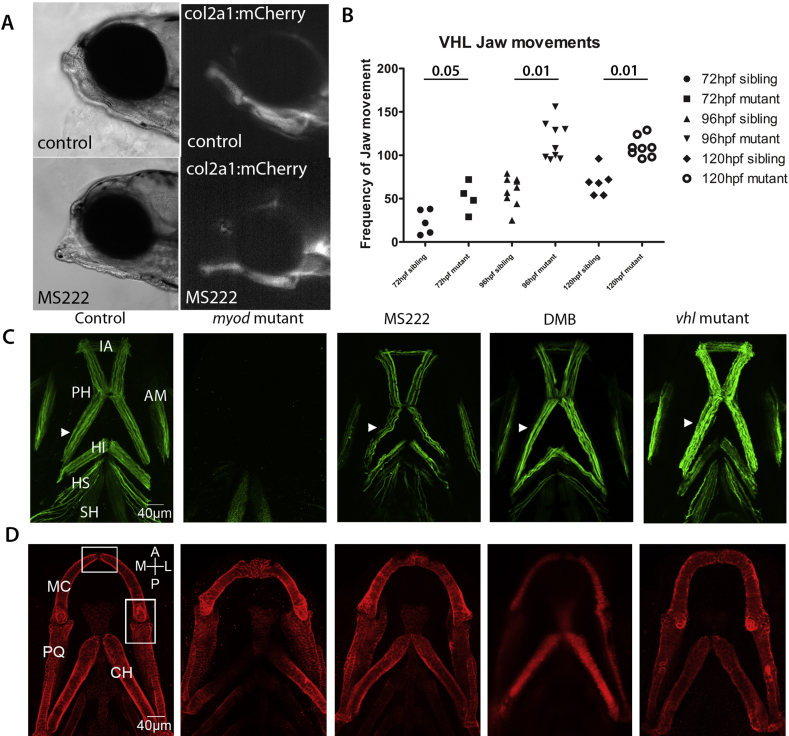
Alterations to movement influence craniofacial skeleton development. A) Flaccid paralysis leads to subluxation of the jaw joint. Brightfield (left hand panels) and Fluorescent images (right hand panels) of lateral views of the head (positioned anterior to left) of 5 day old zebrafish treated with DMSO (top) or MS222 to induce flaccid paralysis (lower panels). Note jaw ‘hanging open’ in MS222 treated larvae due to ‘subluxation’ of the jaw joint. B) Graph showing number of movements per minute in 72, 96 and 120 hours post fertilisation (hpf) sibling and *vhl* mutant larvae (*n* = 4,4,8,9,6,8). Statistical test is a 2 tailed Student *t* test. C) 5dpf craniofacial muscle. A41025 pan-skeletal muscle antibody stain of craniofacial muscle in 5dpf control, *myod* mutant, 3-5dpf MS222 anaesthetic treated DMB treated and *vhl* mutant zebrafish. White arrows mark the muscle fibres of the PH. IA, intermandibularis anterior; PH, protractor hyoideus; AM, adductor mandibularis; HI, hyoideus inferior; HS, hyoideus superior; SH, sternohyoideus. D) Muscle paralysis and hyperactivity affect cartilage jaw morphology. Confocal images, oriented anterior to top, of 5dpf cartilage elements of the lower jaw. 5dpf control, *myod* mutant, 3-5dpf MS222 anaesthetised treated and *vhl* mutant zebrafish cartilage elements were labelled with Collagen II antibody and DMB treated zebrafish cartilage were visualised using the *Tg(Col2a1aBAC:mcherry)* transgenic line. MC = Meckel's cartilage, PQ = palatoquadrate, CH = ceratohyal A, anterior; P, posterior; M, medial; L, lateral. (white boxes show regions for which further images are shown in Fig. 2, Fig. 4).

Flaccid paralysis, in the case of the *myod* mutant and following treatment with MS222 leads to the lower jaw ‘hanging open’ in the majority of cases, such that the jaw joint is subluxed, [Fig. 1(A)]. As expected, larvae subjected to rigid paralysis by DMB maintained a tightly closed jaw throughout (data not shown).

We also wanted to test the impact of increased motility on the joint using 4-amino-pyridine (4-AP). While treatment with 4-AP at doses of 0.5 mM reliably induced hyperactivity measured both by swim motility and frequency of jaw movement for up to 6 h [Sup. Fig. 3(A)–(C)], treatment for longer than 6 h at 0.5 mM led to decreased swim motility, twinned with abnormal appearance of the fish even at lower doses [Sup. Fig. 3(D) and (E)]. We, therefore, concluded that long-term 4-AP treatment led to physiological changes that were not due to a direct effect on the skeleton.

We, therefore, made use of a zebrafish *vhl* mutant line previously reported to display increased frequency of jaw movements. We quantified the number of jaw movements in *vhl* mutants and compared these to controls. *Vhl* mutants move their jaws significantly more than control fish by 3 dpf, with the increased frequency of movement sustained at 4 and 5dpf [Fig. 1(B)]. We also tested the range of motion in *vhl* mutants (maximum displacement 48 μm *n* = 3) and found that although the average value was higher than controls, it lay within the normal range of movements measured in control fish and was not significantly different (Average maximum displacement 38.5 μm from a range of 30.5 μm-57 μm *n* = 5, *P* = 0.0979).

To test whether the different conditions led to an alteration in muscle fibre development or configuration, we stained 5dpf larvae for skeletal myosin [Fig. 1(C)]. At 5dpf the muscles that attach the lower jaw are the PH, the AM, the intermandibularis anterior (IA), the hyoideus superior (HS) and inferior (HI), with the sternohyoideus (SH) located more posteriorly. Functionally, the PH depresses the mandible leading to mouth opening while the AM closes the mouth^42^, ^43^. The basic muscular configuration was similar in all mutants/treatments with the exception of the *myod* mutant, which as previously described lacks all lower jaw musculature except the sternohyoideus [Fig. 1(C)]^34^. We, therefore, consider the *myod* mutant to be an extreme form of flaccid paralysis. Visible differences can be seen in the muscle fibres themselves, the fibres in the MS222-treated larvae appear ‘baggy’, whereas, by contrast, fibres in the DMB-treated larvae appear tauter, and muscles in *vhl* mutants appear enlarged [Fig. 1(C)].

**Fig. 2:**
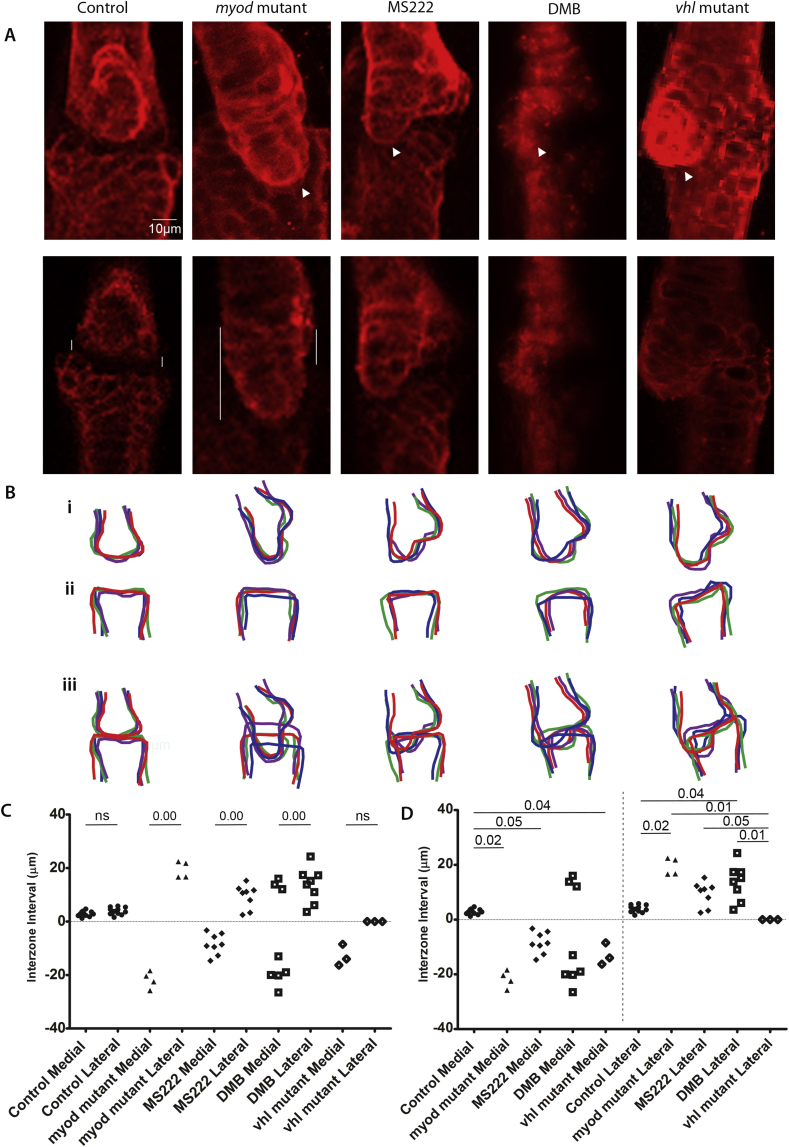
Muscle paralysis and hyperactivity affect cartilage jaw joint morphology. A) Confocal images of 5dpf zebrafish jaw joints. 5dpf control, *myod* mutant, 3-5dpf MS222 anaesthetised treated and *vhl* mutant zebrafish joints were labelled with Collagen II antibody and DMB treated zebrafish cartilage joints were visualised using the *Tg*(*Col2a1aBAC:mcherry*) transgenic line. Max projections are shown in the top panel and single z planes of the jaw joint are shown in the lower panel. White arrowheads show the MC overlapping the PQ element. Vertical white lines mark the medial and lateral interzone interval or extent of element overlap between the MC and PQ elements of the jaw joint as measured. Measurements were consistently taken from a mid joint z plane. B). Representative outlines of the 5dpf jaw joint for each condition (*n* = 4). Meckel's cartilage jaw joint element (Bi), palatoquadrate jaw joint element (Bii) and both elements to show the interaction between the MC and PQ at the jaw joint (Biii). The difference between the interzone interval (μm), on the medial and lateral side of the jaw joint for 5dpf control (*n* = 13), *myod* mutant (*n* = 4), MS222 treated (*n* = 8), DMB treated (*n* = 8) and *vhl* mutant (*n* = 3) compared in (C). The medial and the lateral interzone intervals are compared between each condition in (D). M, medial; L, lateral. ns = not significant, Significance = *P* ≤ 0.05.

To test the impact of changes to movement to the musculoskeletal structure of the lower jaw, we visualised the cartilage structures of the lower jaw by immunostaining for type II collagen [Fig. 1(D)]. We saw, as previously reported that flaccid paralysis (MS222) or lack of muscle (*myod* mutants) showed altered jaw morphology such that the MC overlapped the palatoquadrate (PQ) on the medial side [Figs. 1(D) and 2(A) and^24^]. Interestingly both the rigidly paralysed (DMB-treated) larvae and the hyperactive *vhl* mutants also showed alterations to joint morphology.

Higher magnification images of the joint region [Fig. 2(A) (top panels: max projection) and 2A (bottom panels: single z-plane)] revealed that while control larvae have a complementary shape between the MC and the palatoquadrate with an evenly sized interzone between the two elements, all other conditions showed abnormalities. The muscle-less *myod* mutants, the flaccidly paralysed MS222 treated and the rigidly paralysed DMB-treated larvae all showed a failure to align the cartilage elements such that there was an overlap on the medial surface of the joint and an enlarged gap at the lateral edge [Fig. 2(A) and (B)]. The *vhl* mutants also showed a subtle joint morphology in which there was little visible separation between the MC and the palatoquadrate elements. To quantify these changes we measured the interzone interval on the medial and lateral extremes of the joint [Fig. 2(A) and (C)–(D)]. In control and *vhl* mutant larvae there is no significant difference between the gaps on the medial and lateral sides of the joint [Fig. 2(A) and (C)]. By contrast, all immobilised larvae show significant differences in gap sizes on the medial and lateral sides of the joint, such that the MC overlaps the PQ on the medial side, leaving an enlarged gap on the lateral side [Fig. 2(C)].

**Fig. 3:**
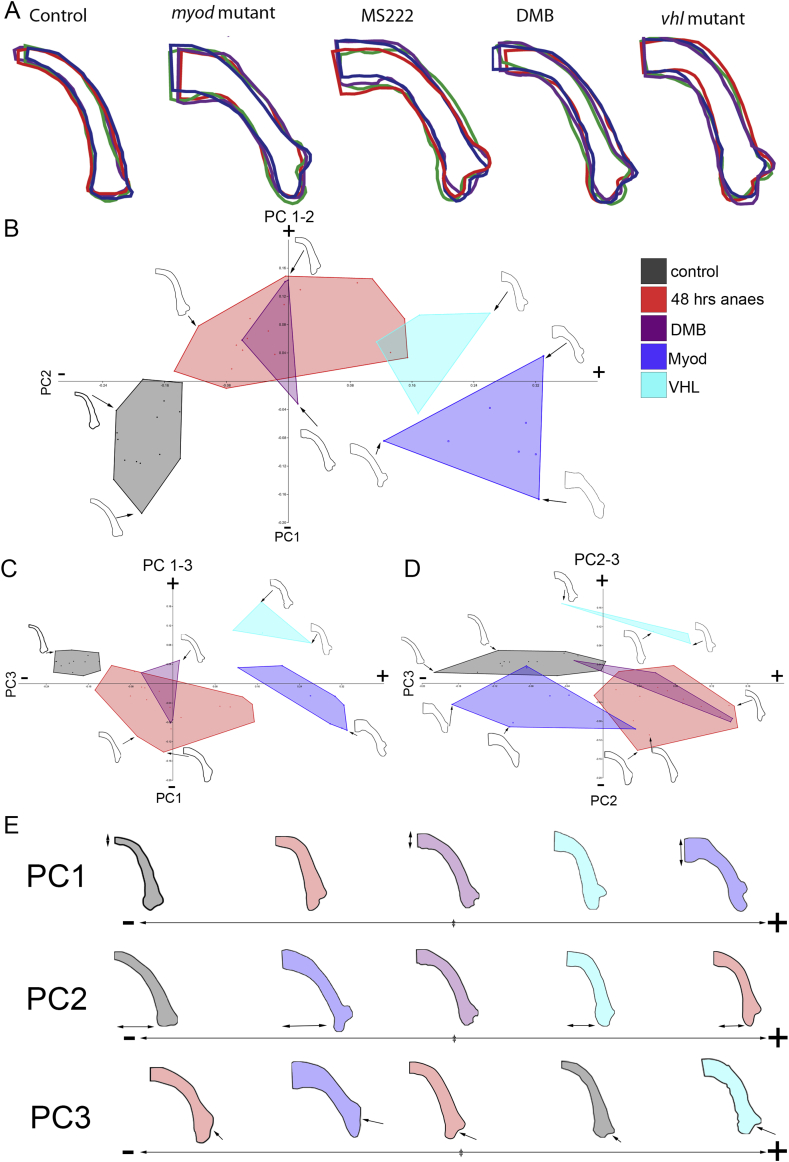
Cartilage element outlines show the variation in cartilage morphology in muscle paralysis and hyperactivity models. A) Representative outlines of the MC element in 5dpf control, *myod* mutant, 3-5dpf MS222 anaesthetised treated, DMB treated and *vhl* mutant zebrafish (*n* = 4 per condition). B) Morphospace plot of PC1 vs PC2, C) PC1 vs PC3, D) PC2 vs PC3 colour coded by treatment group. Each point on morphospace represents one specimen with example outlines for selected specimens indicated with arrow (B–D). E) Simplified representations of the range of PC1, 2 and 3 with selected examples showing the increasing thickness of the MC (PC1, double arrow), decreasing width of the MC (PC2, double arrow) and the overlapping medial surface of the joint (PC3, arrow).

Outlines of the region allow this overlap to be visualised, these outlines demonstrate that the MC shape is more plastic than the PQ [Fig. 2(B)]. To make a fuller assessment of the shape of the whole MC we converted each outline [Fig. 3(A)] into 200 landmarks. These were transformed using the Hangle Fourier transformation and the Procrustes superimposition analysed using a non-parametric manova (npManova) (Table I) and between groups Principle Component Analysis (PCA) (PAST 2.17) (Fig. 3). Results of the npManova (Table I) revealed the MC of drug treated groups and *vhl* and *myod* mutants were significantly different from controls. PCA was used to describe the variation in MC shape. Each principal component (PC) captures a trend in the variation of MC shape, for example, PC1 describes 70.6% of the variation between the groups [Fig. 3(E)] and separates the groups by the increasing width of the MC at the midline (double arrow) from the narrow shape in controls to the wider *myod* and *vhl* mutants on the positive side. PC2 captures 13.9% of the variation and describes the differences in the angle of the arch between controls and the more tightly angled *vhl* mutants and MS222 treated larvae [Fig. 3(E) double arrow]. The overlap in the medial side of the MC is captured by PC3 and accounts for 10.2% of the variation; while PC4 accounts for the final 5.14% of the variation. We generated ‘morphospace’ plots of the shapes represented by these components to explore the similarities and differences between the changing MC shape [Fig. 3 (B)–(D)]. The MS222 and DMB treated groups co-localised in PCs 1–2, 1–3 and 2–3 [Fig. 3 (B)–(D)], in keeping with the results of the non-parametric Manova (npManova) (Table I). The drug treated groups occupy a position along PC1 between the thinner controls and overgrown MC of the *myod* and *vhl* mutants. *Myod* mutants have a narrower arch falling on the negative side of PC2 [Fig. 3(B) and (E)]. Both the *myod* and drug treated groups have an altered medial side (PC3) [Fig. 3(C), (D), (E)]. Significant differences can be seen between *vhl* mutants and flaccid paralysis, but not between hypermobility and rigid paralysis (*P* = 0.29, Table I).

**Fig. 4:**
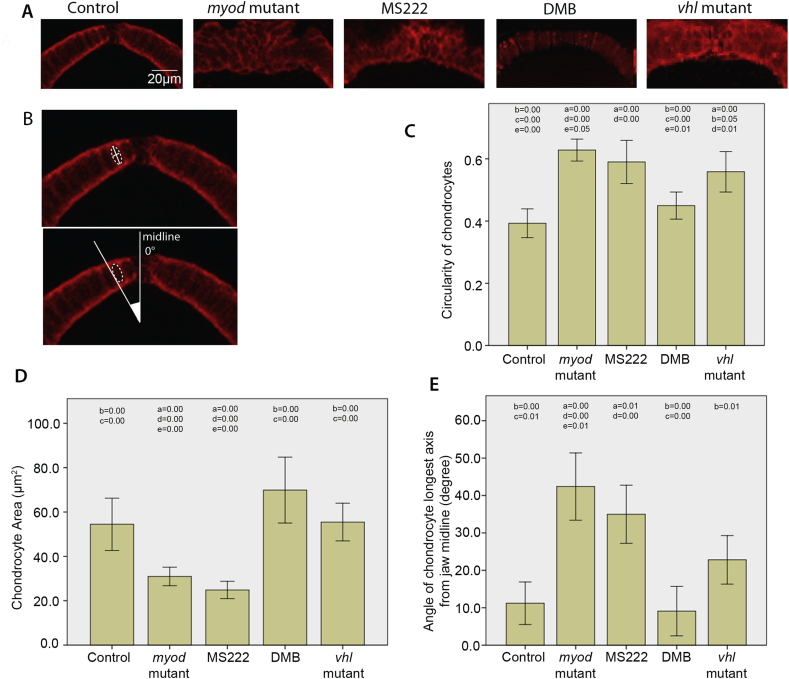
Flaccid paralysis affects the maturation of chondrocytes at the anterior tip of the MC. A) Confocal images of the anterior MC. 5dpf control, *myod* mutant, 3-5dpf MS222 anaesthetised treated and *vhl* mutant zebrafish cartilage was labelled with Collagen II antibody and DMB treated zebrafish cartilage was visualised using the *Tg(Col2a1aBAC:mcherry)* transgenic line. B) Dotted white lines show example cell. Solid white lines mark an example major and minor axis of a collagen II labelled chondrocyte used to calcuate the circularity of the cell (top panel), white lines display angle of cell relative to the midline (bottom panel). C) Graph showing comparison of cell circularity of chondrocytes at the tip of the MC in 5dpf control (*n* = 30), *myod* mutant (*n* = 33), 3-5dpf MS222 anaesthetic treated (*n* = 26), DMB treated (*n* = 28) and *vhl* mutant zebrafish (*n* = 33) (N.B 1.00 represents a perfect circle). D) Chondrocyte area measurements from cells at the tip of the MC in 5dpf control (*n* = 29), *myod* mutant (*n* = 28), 3-5dpf MS222 anaesthetic treated (*n* = 34), DMB treated (*n* = 22) and *vhl* mutant zebrafish (*n* = 31). E) Comparison of angle of the major axis of cell relative to the midline in 5dpf control (*n* = 9), *myod* mutant (*n* = 41), 3-5dpf MS222 anaesthetic treated (*n* = 47), DMB treated (*n* = 10) and *vhl* mutant zebrafish (*n* = 34). For all graphs at least 3 fish were measured per condition with at least 9 cells equally spaced between the insertion points of the intermandibularis muscle measured per fish. *P* values that are less than or equal to 0.05 between conditions are marked on the graph (in correspondence to the following key), to show which conditions are significantly different to one another. a = control, b = *myod* mutant, *c* = 3-5dpf MS222 anaesthetic treated, d = DMB treated, e = *vhl* mutant zebrafish. Graphs show 95% confidence intervals.

Morphospace analysis revealed differences to the morphology of the anterior tip of the MC. We took high magnification images of this region (Fig. 4), which showed differences between the behaviour of cells in this region; such that cells in flaccid paralysis and muscle-less mutants appeared small and rounded, whereas cells in DMB-treated fish appeared similar to controls with an enlarged, elongated shape; suggestive of increased maturity [Fig. 4(A)]. To test this we measured cell ‘circularity’, taking the ratio between the cell's major and minor axis [Fig. 4(B) and (C)]. This showed cells from *myod* mutants and anaesthetised fish were significantly more rounded than controls; whereas DMB-treated fish did not differ from controls [Fig. 4(C)].

Another measure of maturity is increased cell volume. We tested the cell area [Fig. 4(D)]; *myod* and flaccidly paralysed fish had significantly smaller cells than controls, whereas, cell size in *vhl* mutants and the DMB-treated fish did not significantly differ from controls [Fig. 4(D)].

Finally, another sign of maturity is cell intercalation as they stack into the mature cartilage shape. We, therefore, measured the angle of cells at the MC tip and found while anaesthetised and *myod* mutants had significant differences to cell orientation to controls, rigidly paralysed and hypermobile larvae did not [Fig. 4(E)].

**Fig. 5:**
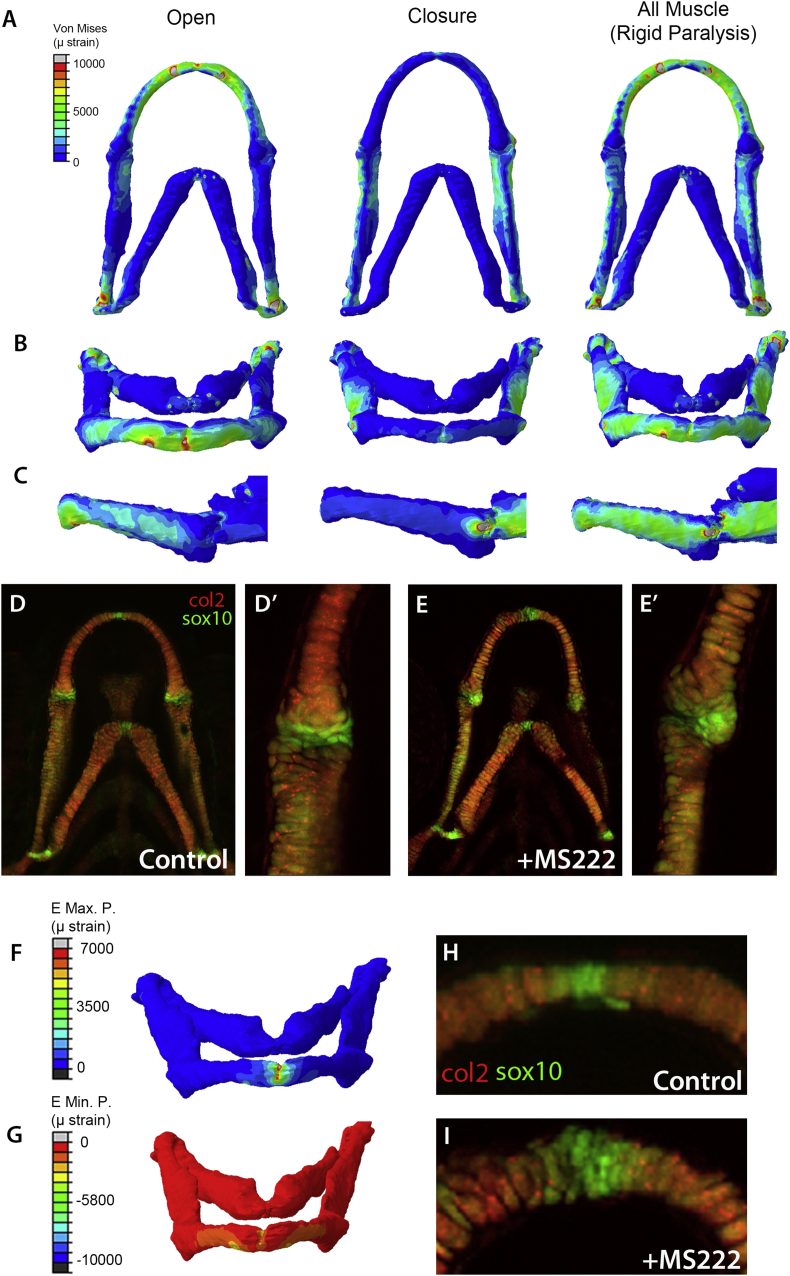
Biomechanical strains correlate with sites of cell maturation. Finite Element models showing locations of Von Mises strain (in microstrain) during mouth opening (IM and PH muscles are applied), closure (AM muscles applied) and in situations where the IM, AM and PH muscles are applied simultaneously to replicate rigid paralysis (A–C). Ventral view (A), Frontal view (B) and Lateral view (C). Confocal stacks of the ventral jaw and jaw joint in the double transgenic line col2a1:mcherry (red) and sox10:eGFP (green) in 5 dpf control zebrafish (D, D′) and immobilised (E,E′) zebrafish. Cells in yellow are seen where the two trangenes are coexpressed. Green cells (which are sox10+ve, col2-ve and therefore less differentiated) are concentrated in the joint regions and in the anterior tip of the MC. Note the altered distribution in the immobilised fish which corresponds to the shape changes observed. Finite Element models showing locations of Maximum principal strain [tension] (F) and Minimum principal strain [compression] (G) in the anterior tip of the MC when the IM muscle alone is applied. Confocal images of the MC anterior tip region (H,I) showing expansion of the distribution of sox10 positive cells in MS222 treated larvae (I) relative to controls (H).

To explore the alterations to mechanical environment of the cells in the MC we generated Finite Element (FE) models for jaw opening and closure and to replicate rigid paralysis [Fig. 5 (A)–(C)]. These models show that muscular strain is concentrated around the tip of the MC during jaw opening and in rigid paralysis [Fig. 5 (A)–(C)]. High strains are located at the joint regions during jaw closure and during rigid paralysis. The most parsimonious explanation for the alterations to cell size and morphology at the MC tip in flaccidly paralysed larvae and the difference between flaccid and rigid paralysis is that the ‘small, round’ cells at the MC tip are less differentiated than the larger and more polarised cells that have intercalated in the wild type and DMB-treated fish. To test this we used the transgenic reporter, sox10:eGFP. Sox10 marks all migratory neural crest precursors and therefore, marks cells of the interzone and precursor cells, which form part of the element prior to expression of type II collagen^36^. We crossed *sox10:GFP* with the *col2a1:mCherry* line, in this cross cells which are less differentiated are green or pale yellow whereas more mature cells will be orange or red. We imaged the ventral jaw of control and MS222 immobilised fish at 5dpf [Fig. 5 D,D′,E,E′,H,I]. In control fish the regions which contain less mature cells correspond to joints, including the jaw joint between the MC and PQ, to the tip of the MC and between the ceratohyal elements [Fig. 5(D),(D′),(H)]. In these positions we observed differences in the MS222 treated larvae [Fig. 5(E),(E′),(I)]. We observed changes to the number, and distribution of strongly sox10 positive cells in the joint between the MC and PQ [Fig. 5(D)′,(E′)], suggesting changes to the maturation of precursor cells in the joint. To explore the mechanics operating at the tip of the MC we generated FE models in which the IM muscle alone was applied to test whether biomechanical strain patterns from this muscle could explain the differences to cell behaviour between flaccidly and rigidly paralysed larvae [Fig. 4(C)–(E)]. Application of the IM alone leads to relative high maximum principal strain (tension) centred on the tip of the MC [Fig. 5(F)], with relatively little minimum principal strain (compression) in this region [Fig. 5(G)]. The positions in which high strains are seen upon application of the IM muscle overlap those in which cell behaviour is altered when paralysed. Correspondingly, we see increased numbers of immature cells marked by expression of *sox10* but not *col2a1* in this region in flaccidly paralysed fish compared to controls [Fig. 5(H),(I)].

Therefore, the differences in cell morphology and therefore element shape at the anterior of the MC between rigid and flaccid paralysis can likely be explained by the requirement for tension from contraction of the IM.

## Discussion

We have previously shown that lack of jaw muscle activity in flaccidly immobilised larvae leads to altered joint shape^24^. In this paper we explored the differences and similarities in skeletal development under continuous or absent muscle load compared with control and hyperactive zebrafish.

Flaccid and rigid paralysis of zebrafish caused similar changes to the morphology and function of the jaw joint. However, in fish as in chicks, there were overall differences between flaccid and rigidly paralysed skeletal morphologies. In chicks flaccid paralysis led to a wider range of phenotypes including abnormal cartilage morphology, which we also see^29^. In fish, these morphological abnormalities were most pronounced at the tip of the MC. In the presence of dynamic muscle force, chondrocytes at the tip of the MC in control zebrafish have an ordered structure and the jaw joints develop correctly. In the absence of load (MS222 treated and *myod* mutants) the chondrocytes remain immature as indicated by the large number of sox10 positive cells and fail to orient correctly at the tip of the MC. The jaw joints also have a characteristic overlapping morphology. Under rigid paralysis (DMB-treated) where muscle are continuously contracting the orientation of the chondrocytes at the tip of the MC were not significantly different from control and appear mature. However, the general shape of the MC and the joints were altered. This suggests that dynamic movement, rather than muscle forces are required for normal joint morphogenesis, whilst successful chondrocyte maturation at the tip of the MC requires only the presence of muscle force. Our examination of the mechanics through FE modelling strongly suggests that tension exerted by contraction of the intermandibularis muscle provides a mechanical stimulus for the chondrocytes in this region to mature. This explains both the changes to cell morphology and the increased expression of sox10 relative to col2a1, a marker of mature chondrocytes, in immobilised zebrafish.

We also show the effects of hyperactivity on skeletal development. Interestingly, while hyperactivity led to altered skeletal behaviour; generally the effects were less dramatic than those elicited by paralysis. This suggests that movement and force are required to shape skeletal development and that normal levels of activity are optimal^18^, ^29^. We show in *vhl* mutants that although the frequency of movement is approximately twice that of controls their range of jaw movement was normal. Perhaps if the range of motion had differed this might have a more severe impact on joint morphology, in monkeys surgical hyperextension of the jaw led to significant alterations to cartilage morphology^44^, ^45^. One way in which our hypermobility results differ from those in chicks is that we show the size of the interzone is diminished in hypermobile *vhl* mutants, whereas in chicks hypermobility increased joint cavity size^18^, ^29^. These differences in joint structure may be explained by the timing of hypermobility as our experiments are all undertaken at precavitation stages, or alternatively the effect of hypermobility on joint structure may differ between species.

Taken together the data suggest that dynamic mechanical forces control cartilage element morphology and joint shape by controlling a number of cellular behaviours, including proliferation, orientation, migration and differentiation and that these differ dependent on their local mechanical forces. This fits with *in vitro* literature that shows different mechanical forces can differentially regulate markers of chondrocyte maturation, with tensile strain pushing cells towards maturation, while hydrostatic pressure slowed chondrocyte maturation^46^, subsequent work in stem cells has demonstrated that hydrostatic pressure stabilises the chondrocyte phenotype preventing entry to hypertrophy^47^. In this context it suggests that tension at the Meckel's tip is required for chondrocyte maturation in this region, while different forces resulting from dynamic movement prevent premature maturation of interzone cells, maintaining them in a state where they retain the ability to migrate or reorient, allowing optimal joint morphology to be sculpted by movement so that joint surfaces are complementary. We have previously shown that joint cells in flaccidly paralysed larvae are incorrectly oriented^24^, the prediction from these data would be that rigidly paralysed larvae would also lead to abnormal cell orientation at the joint due to the lack of dynamic movement during paralysis.

Recently there have been significant efforts to identify mechanosensitive genes from the skeletal system; including the correlation of gene expression patterns to mechanical stimuli in chick limbs^14^, ^25^, and transcriptomics to identify all genes that show significant changes to expression in the humerus of control and muscle-less mice^26^, ^48^. Human GWAS have also identified a number of genetic associations with joint geometry^49^, ^50^. However, it remains difficult to test the functional effects of gene manipulation in a model system. Zebrafish have the advantage of being genetically tractable, therefore, a better understanding of the dynamic process of joint morphogenesis should in future allow the functional testing of putative mechanosensitive genes to test their ability to elicit certain cell behaviours.

## Author contributions

The study was designed by CLH. REHS, LHB, KAR and NMA performed experiments. All authors helped in data analysis and in drafting and reviewing the manuscript.

## Conflicts of interest

The authors report no conflicts of interest. All animal work was subject to internal ethical approval and Home Office regulations.

## Figures and Tables

**Table I:** Modifications to jaw movement caused by paralysis and the vhl or myod mutations cause significant changes to the shape of the MC

	Control	myod	Flaccid MS222	Rigid DMB	Hyper Vhl
Control	–	0.001	0.001	0.001	0.006
myod	0.001	–	0.001	0.019	0.087
Flaccid MS222	0.001	0.001	–	1	0.001
Rigid DMB	0.001	0.019	1	–	0.29
Hyper vhl	0.006	0.087	0.001	0.29	–

## Appendix A Supplementary data

Supplementary Figure 1 Measurement maximum jaw displacement. A brightfield video of jaw movement (Sup. Vid. 2) is exported into ImageJ. The two most extreme positions of the anterior tip of the MC (black arrowhead) from separate video frames are found and marked (black dots). The lower dot corresponds with the position of the MC of the current frame and the upper dot corresponds with the position of the MC at maximum closure from a previous frame. Maximum jaw displacement is measured in ImageJ between the two points (red line).

Supplementary Figure 2 A) Confocal image of the lower jaw, showing the outline collected for morphospace analysis. B) Outline generated showing the origin.

Supplementary Figure 3 A) Graph showing average velocity of tracked fish treated with doses of 4AP or in the case of control DMSO alone. Tracks were taken from 10 fish per dose and error bars represent 95% confidence intervals. (*n* = 10) B) Graph to show average distance travelled per fish when tracked over a period of 6 minutes treated with doses of 4AP or in the case of control DMSO alone. Tracks were taken from 10 fish per dose and error bars represent 95% confidence intervals (*n* = 10). C) Number of mouth openings per minute in control and fish treated with 0.5mM 4AP for 3 h. Measurements taken from 4 fish per treatment. Statistical test is a 2 tailed students *t*-test *P* < 0.01. (*n* = 4) D) Brightfield image of a control larva at 5 dpf. E) Brightfield image of a representative larva treated with 0.1mM 4AP from 3–5 dpf.
