# Supplementary figures and images for: Differential effects of altered patterns of movement and strain on joint cell behaviour and skeletal morphogenesis
Source: Osteoarthritis Cartilage. 2016 Nov;24(11):1940–50. doi: 10.1016/j.joca.2016.06.015 (PMC5081689; doi:10.1016/j.joca.2016.06.015)

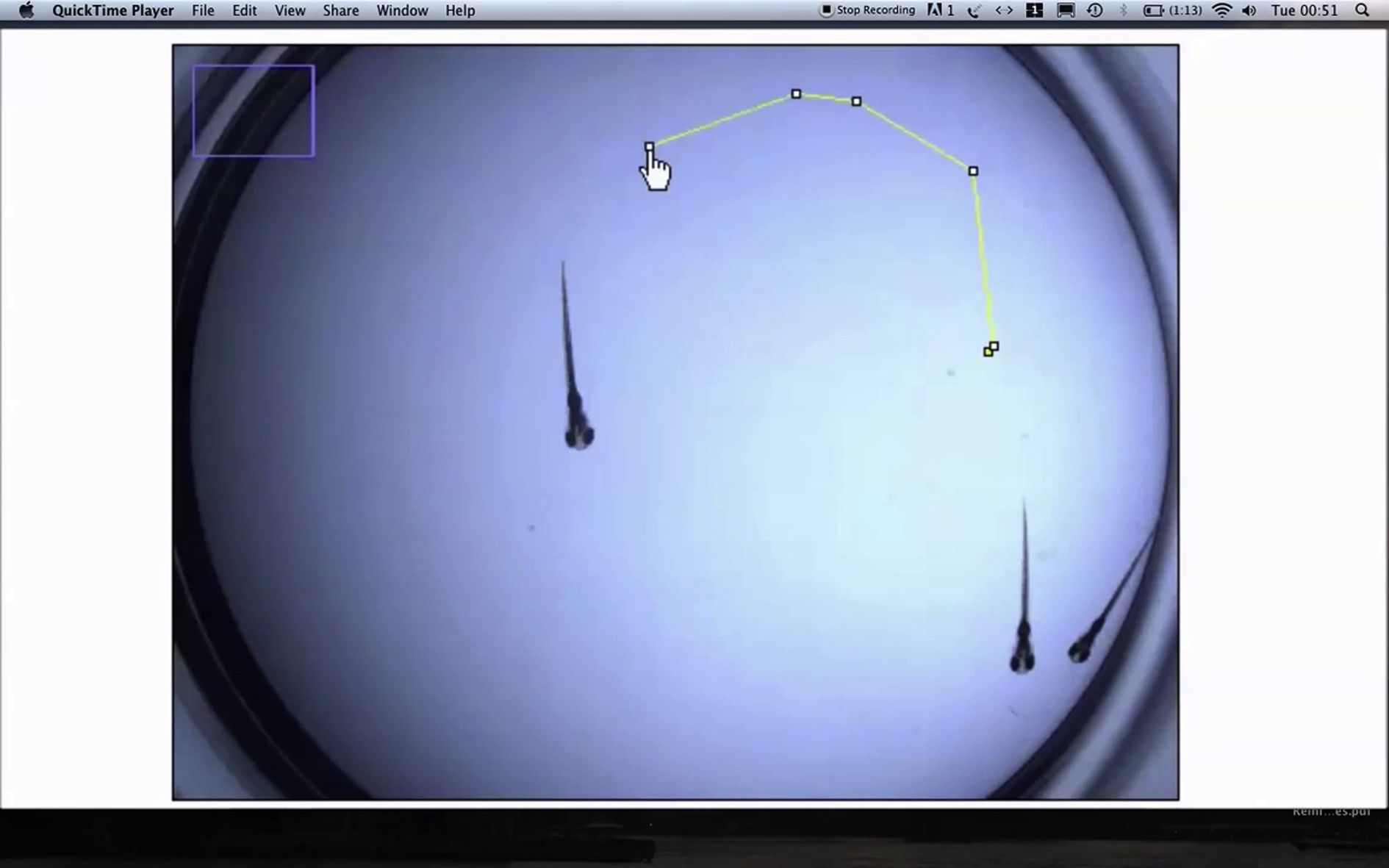

Supplement: Supplementary Video 1 — Zebrafish swim motility tracking. Control and 4AP-treated zebrafish swim motility was recorded in petri dishes. ImageJ software was used to manually track the distance of zebrafish movement in 6 min measured from the end of each larval tail. Velocity of zebrafish movement could then be determined from the distance travelled over time. [file mmc1.jpg]

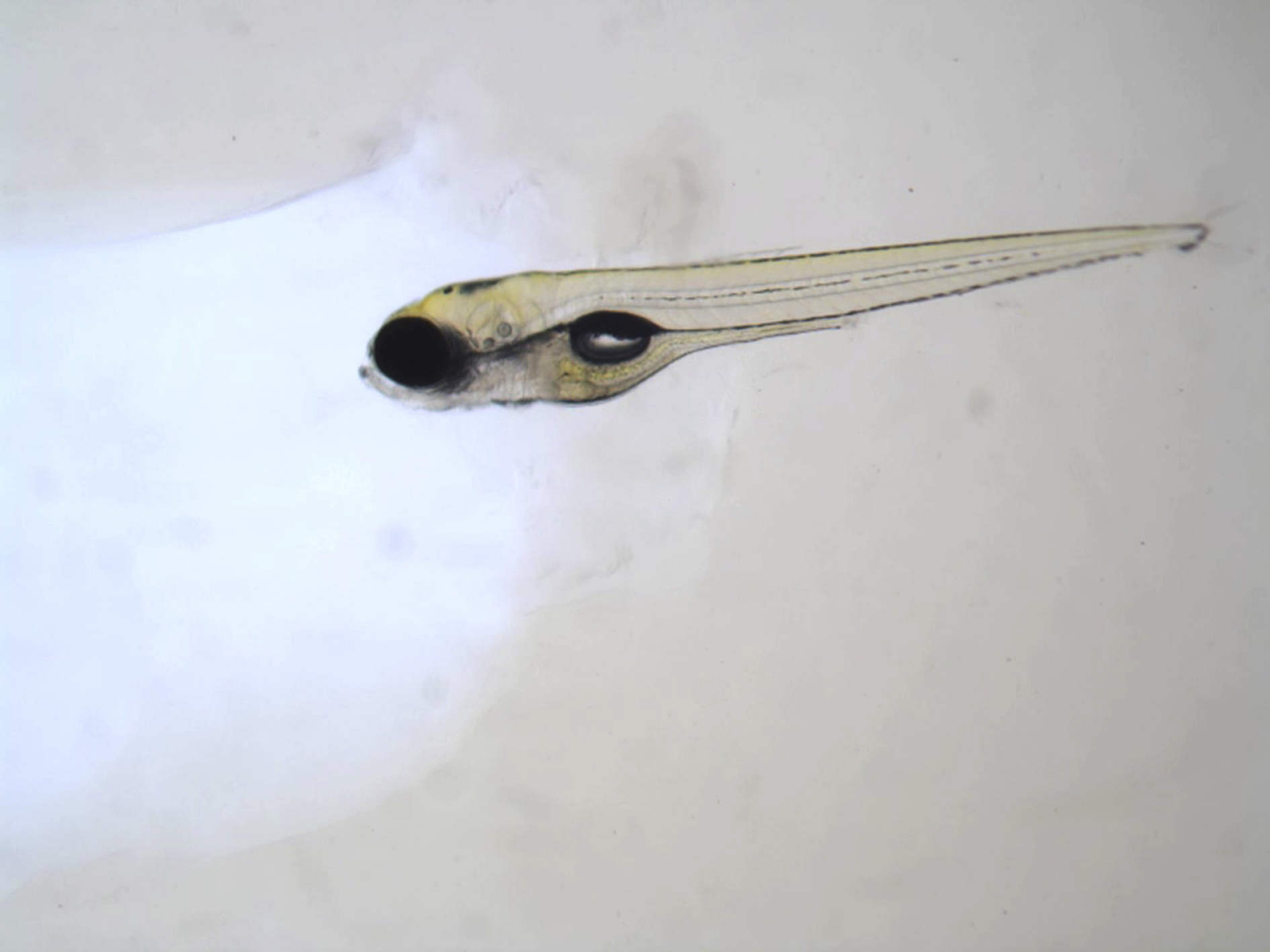

Supplement: Supplementary Video 2 — Recording of frequency of jaw movement. Brightfield video of a 4dpf larva embedded in 1% agarose and bathed in Danieau buffer with agarose removed from around the head to allow for jaw movements. [file mmc2.jpg]

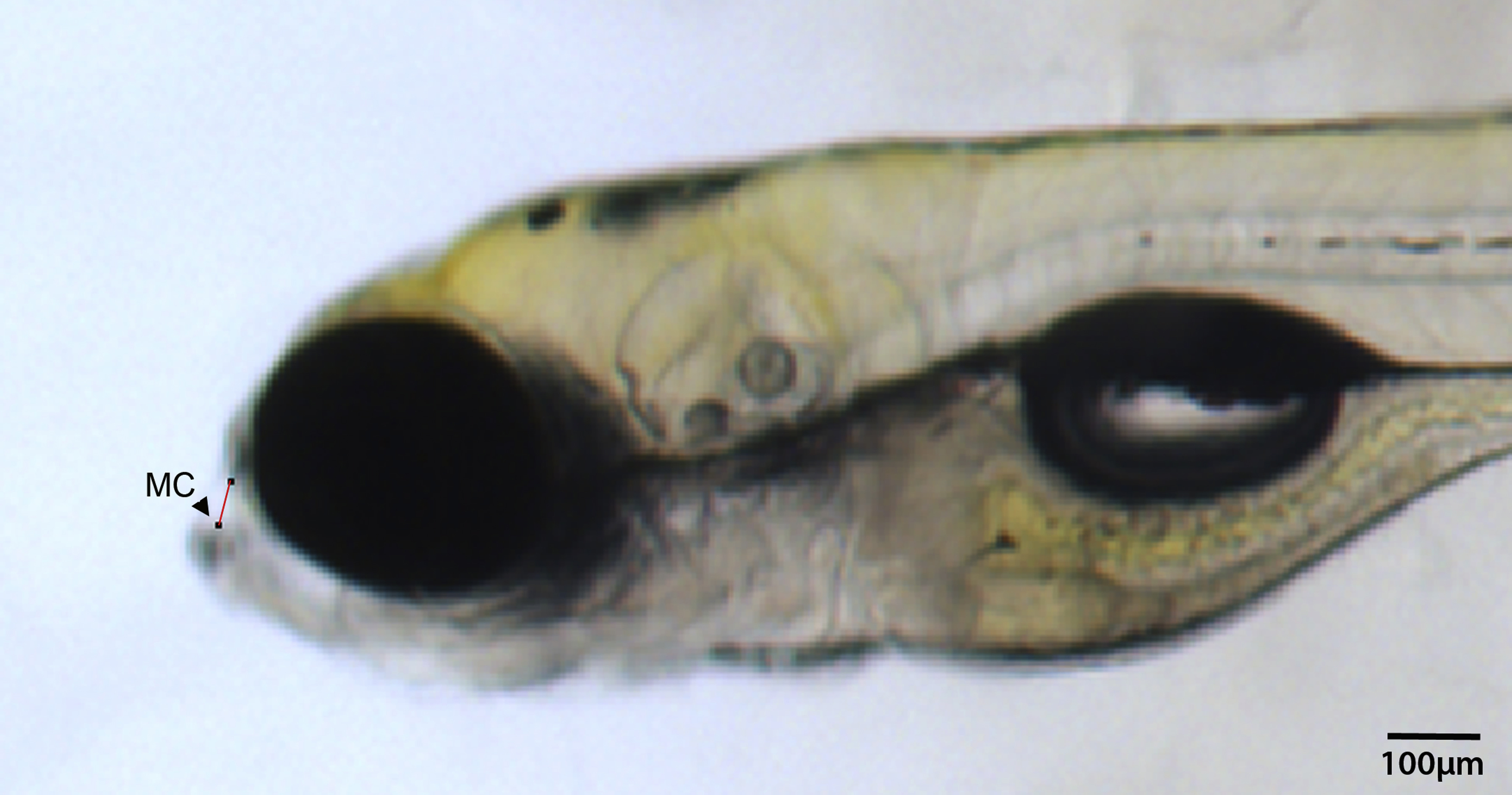

Supplement: Supplementary Figure 1 — Measurement maximum jaw displacement. A brightfield video of jaw movement (Sup. Vid. 2) is exported into ImageJ. The two most extreme positions of the anterior tip of the MC (black arrowhead) from separate video frames are found and marked (black dots). The lower dot corresponds with the position of the MC of the current frame and the upper dot corresponds with the position of the MC at maximum closure from a previous frame. Maximum jaw displacement is measured in ImageJ between the two points (red line). [file figs1.jpg]

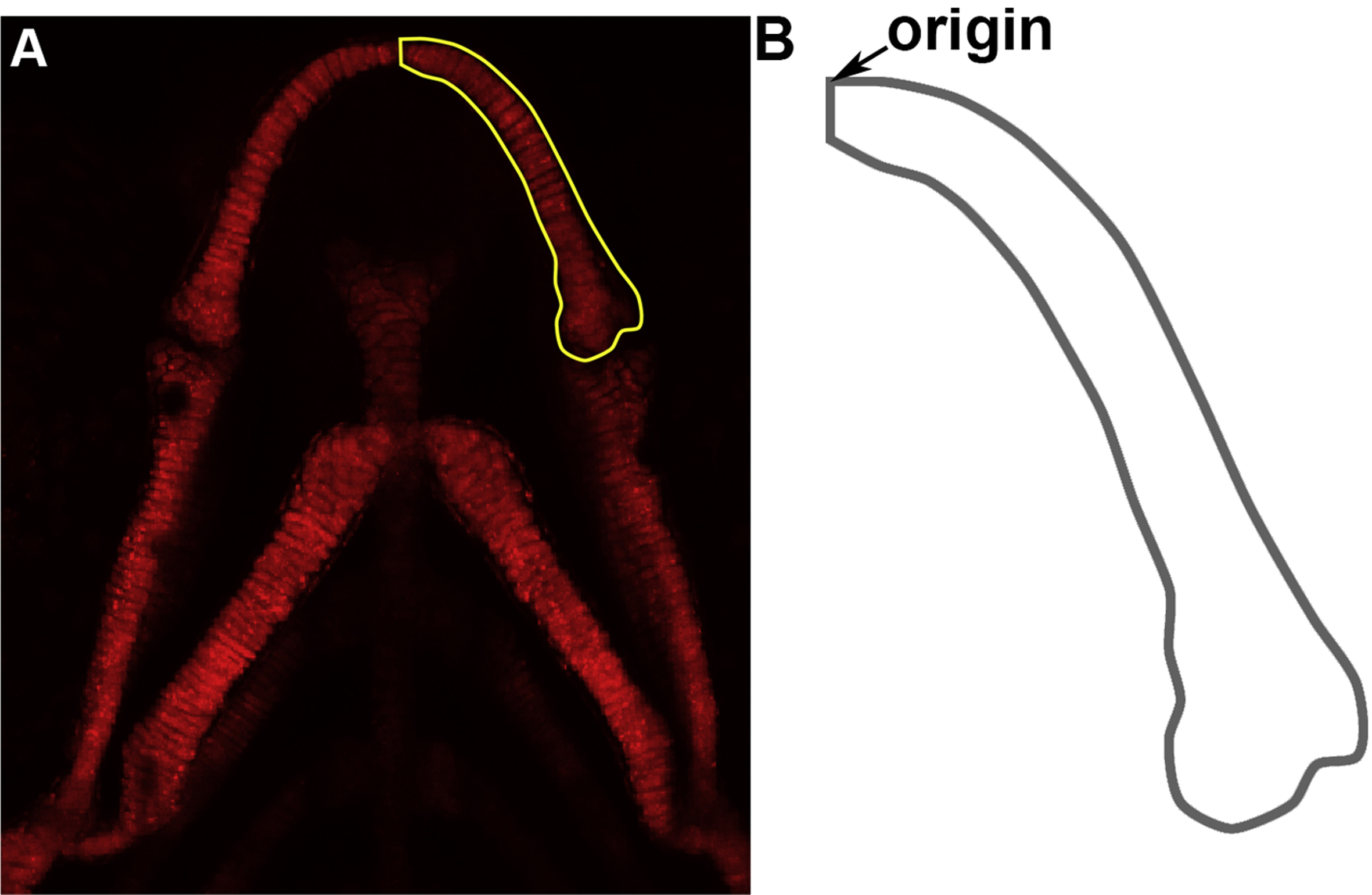

Supplement: Supplementary Figure 2 — A) Confocal image of the lower jaw, showing the outline collected for morphospace analysis. B) Outline generated showing the origin. [file figs2.jpg]

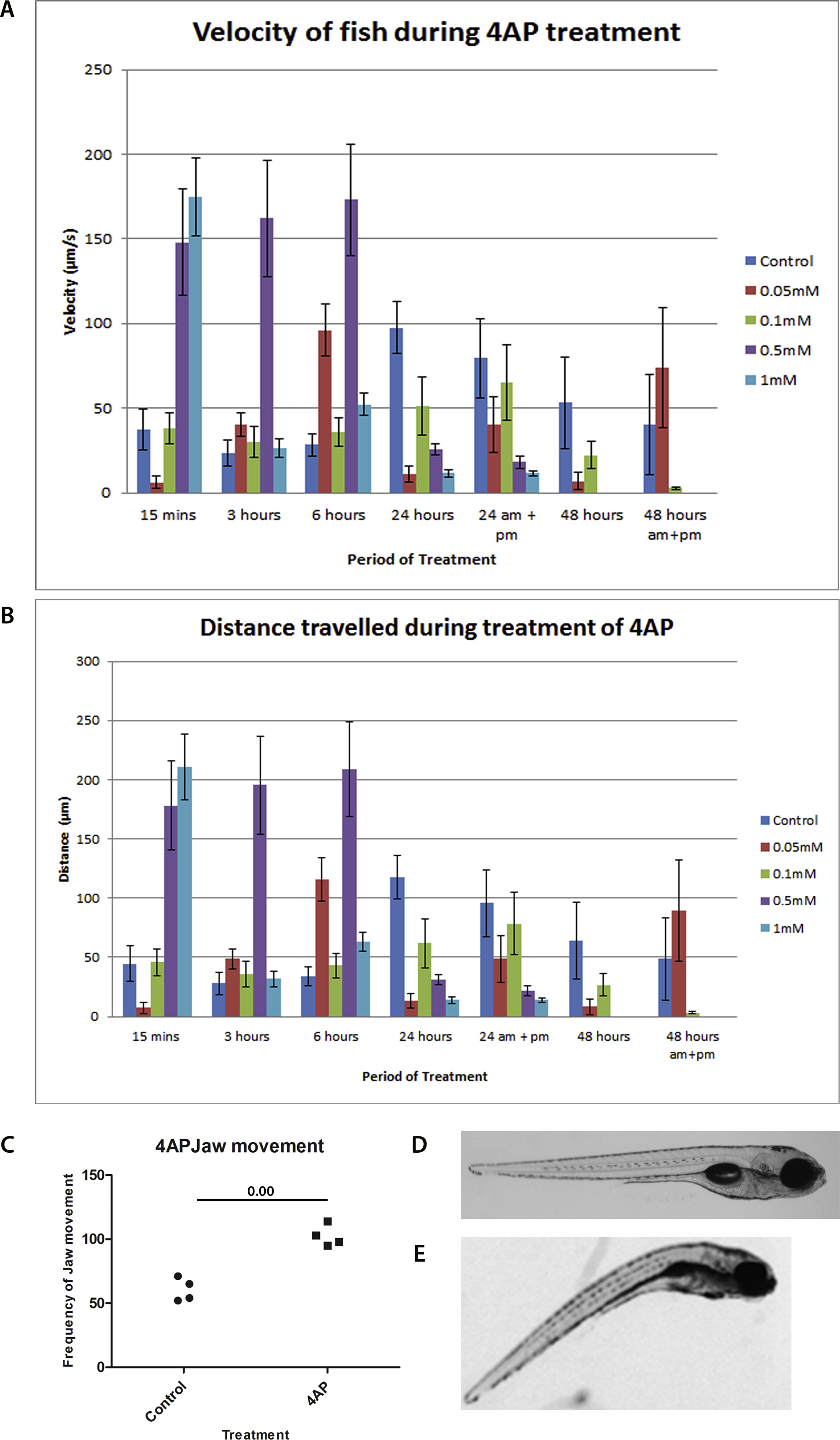

Supplement: Supplementary Figure 3 — A) Graph showing average velocity of tracked fish treated with doses of 4AP or in the case of control DMSO alone. Tracks were taken from 10 fish per dose and error bars represent 95% confidence intervals. (n = 10) B) Graph to show average distance travelled per fish when tracked over a period of 6 minutes treated with doses of 4AP or in the case of control DMSO alone. Tracks were taken from 10 fish per dose and error bars represent 95% confidence intervals (n = 10). C) Number of mouth openings per minute in control and fish treated with 0.5mM 4AP for 3 h. Measurements taken from 4 fish per treatment. Statistical test is a 2 tailed students t-test P < 0.01. (n = 4) D) Brightfield image of a control larva at 5 dpf. E) Brightfield image of a representative larva treated with 0.1mM 4AP from 3–5 dpf. [file figs3.jpg]
